# Supplementary material for: Comprehensive Annotation and Functional Exploration of MicroRNAs in Lettuce
Source: Front Plant Sci. 2021 Dec 24;12:781836. doi: 10.3389/fpls.2021.781836 (PMC8739914; doi:10.3389/fpls.2021.781836)
Supplement: Supplementary file 3 [file Data_Sheet_9.ZIP › GSM2230753.plot/Lsa-miR403_Lsat_1_v5_gn_4_22040.1_3191_TPlot.pdf]

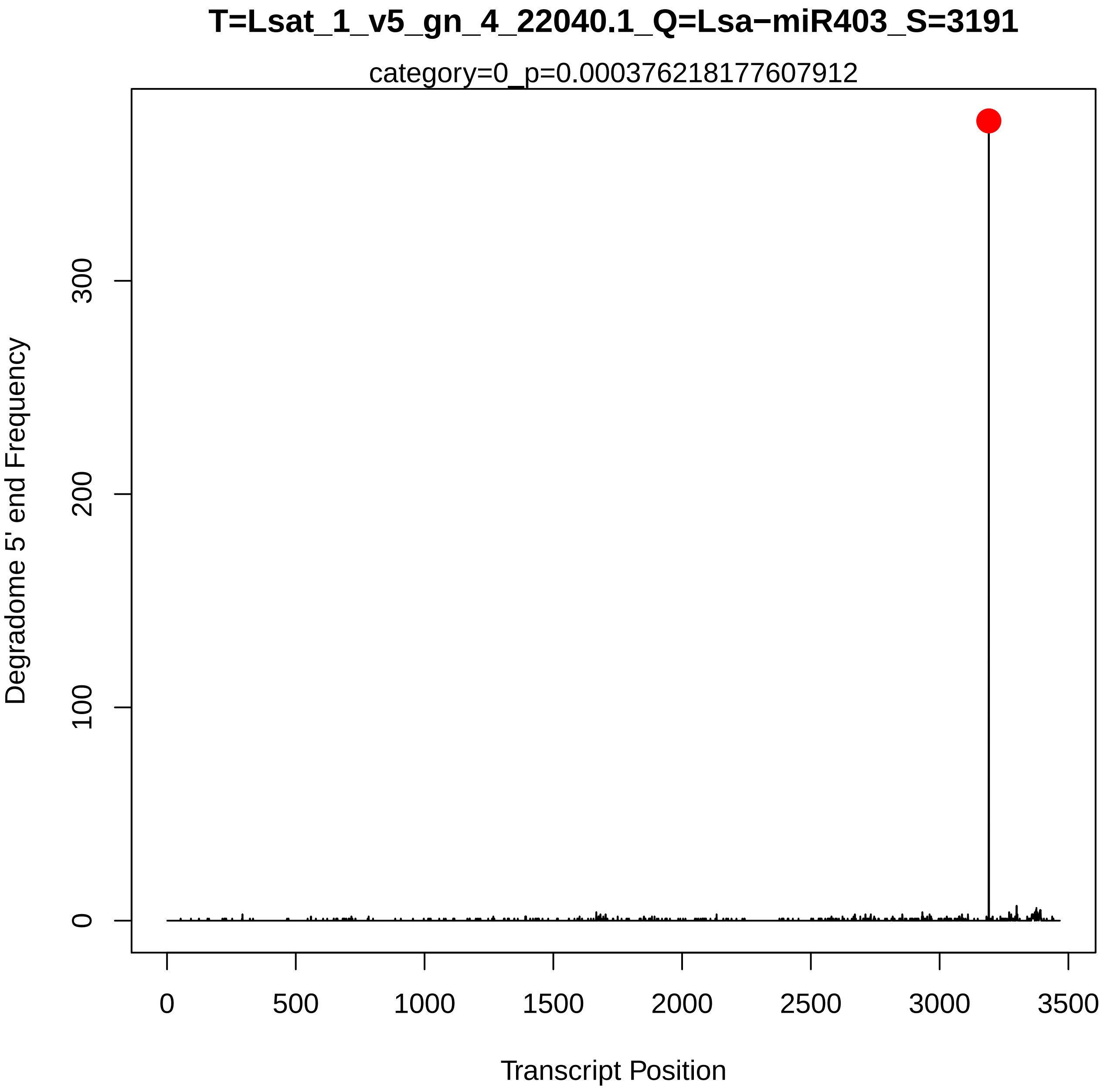

T=Lsat_1_v5_gn_4_22040.1_Q=Lsa−miR403_S=3191
category=0_p=0.000376218177607912
Degradome 5' end Frequency
0
100
200
300
0
500
1000
1500
2000
2500
3000
3500
Transcript Position
